# Epidemiology of Childhood Cancer and Cancer Predisposition Syndromes (CPSs): A 20-Year Single-Center Cohort from the Greater Poland Region

**DOI:** 10.3390/children13060778

**Published:** 2026-06-03

**Authors:** Gabriela Telman-Kołodziejczyk, Adrian Guźniczak, Patrycja Sosnowska-Sienkiewicz, Danuta Januszkiewicz-Lewandowska

**Affiliations:** 1 Doctoral School, Poznan University of Medical Sciences, Bukowska Street 70, 60-812 Poznan, Poland; 2 Faculty of Medicine, Poznan University of Medical Sciences, Fredry 10 Street, 61-701 Poznan, Poland; guzniczak.adrian@gmail.com; 3 Department of Pediatric Surgery, Medical University of Warsaw, Zwirki i Wigury 63A Street, 02-091 Warsaw, Poland; patrycja.sosnowska-sienkiewicz@wum.edu.pl; 4 Department of Pediatric Oncology, Hematology and Transplantology, Poznan University of Medical Sciences, Szpitalna 27/33, 60-572 Poznan, Poland

**Keywords:** pediatric oncology, childhood cancer, cancer predisposition syndromes (CPSs)

## Abstract

**Highlights:**

**What are the main findings?**

• CPS-positive patients were diagnosed with a neoplasm at a significantly younger age than CPS-negative patients; crucial differences were observed for liver and intrahepatic bile duct tumors, myeloid leukemia, lymphoid leukemia, and renal tumors.
• Among children with any kind of blastoma cancer, excluding medulloblastoma, CPS co-occurred in over $\frac{1}{5}$ of cases in each corresponding ICD-10 classification group, representing the most representative group, followed by CPS-positive patients with central nervous system tumors (including medulloblastoma, another kind of blastoma cancer).

**What are the implications of the main findings?**

• Early age of neoplasm onset in children and adolescents should prompt diagnostics for CPSs, as long as they remain underreported conditions. Also, following the international surveillance guidelines for CPS-positive patients is crucial for the early detection of most neoplasms in these children and adolescents.
• To our knowledge, this is the first study to report a higher prevalence of CPSs among patients suffering from blastoma-type cancers. Further research is required, not only at other centers of pediatric oncology, but also nationwide to obtain a more representative group collection.

**Abstract:**

**Importance:** A comprehensive analysis of childhood cancer and cancer predisposition syndromes (CPSs) incidence can provide insights that lead to improvements and modifications in treatment protocols through personalized therapy, thereby reducing toxicity. **Purpose:** This study aimed to analyze age-specific hospital-based childhood cancer rates and the distribution of CPSs in a 20-year pediatric cohort from the region. **Materials:** A total of 2190 patients, aged from birth to 17 years, diagnosed with any type of neoplasm classified by ICD-10 codes at Karol Jonscher’s Clinical Hospital of Poznan University of Medical Sciences (KJCH PUMS) between 1 January 2000, and 31 December 2019, were included, with 193 (8.8%) having an underlying CPS. **Results:** The pediatric population of the Greater Poland Region has declined over the past two decades. The most common diagnoses can be grouped into three main categories: (1) leukemias, involving 704 patients (32.1%); (2) central nervous system (CNS) tumors, represented by 382 children (17.4%); and (3) lymphomas, including 279 patients (12.7%), together accounting for 1353 cases (61.8%). The age-specific hospital-based case rate for childhood cancer (all types combined) peaked in the 0–28 days age group at 71.8 per 100,000 person-years (95% CI: 52.2–96.4), with a trend to decrease with age and a slight increase among adolescents aged 16–17 years (13.6 per 100,000, 95% CI: 12.0–15.4). The age-specific incidence of CPS-positive cancers declined from 18.0 (95% CI: 8.2–29.4) per 100,000 person-years in the first month of life to 0.7 (95% CI: 0.3–1.2) in 16–17-year-olds. CPS-positive children were diagnosed at significantly younger ages for four cancer types: liver and intrahepatic bile duct tumors (C22: A = 0.097, adjusted *p* < 0.001), myeloid leukemia (C92: A = 0.179, adjusted *p* < 0.001), lymphoid leukemia (C91: A = 0.309, adjusted *p* = 0.007), and renal tumors (C64: A = 0.335, adjusted *p* = 0.013). **Conclusions:** CPSs likely play a significant and underestimated role in pediatric cancers, especially during early childhood. Improving access to genetic testing could greatly enhance risk assessment, personalized treatment, and long-term outcomes in pediatric oncology.

## 1. Introduction

As long as cancers in children, although rare compared to adults, remain a leading cause of death in the pediatric population, their early detection and proper, often personalized treatment, remain challenging for modern medicine worldwide [1,2,3]. The integration of genetics, medical engineering, science, and medicine has led to improvements in therapeutic effectiveness over the past few decades [4,5,6,7,8,9,10,11]. A crucial factor enabling the achievement of current treatment outcomes is the collection of patient data in regional and national cancer registries. These registries allow for follow-up and analysis of undertaken therapy, leading to valuable conclusions such as modifications in cancer treatment protocols for specific patient groups (e.g., with gene mutations, stages of disease, or younger age) or for supervised neoplasms in general [12,13,14,15,16], as well as estimating predicted local and global cancer morbidity trends [17,18,19]. Long-term retrospective registry data analyses highlight current and future challenges for medicine by (1) revealing prevalent diseases in different age groups that are most likely to be detected [20,21], (2) diagnosing uncommon cancers among children, which must be considered in differential diagnosis despite their rarity [22,23,24,25] or (3) listing neoplasms with higher risks of death from the primary diagnosis and subsequent chronic health conditions in long-term survivors [26,27,28,29]. Furthermore, they can also demonstrate the scope of medical tourism, both within the country and worldwide [30,31].

The aim of this study was to analyze age-specific childhood cancer rates based on hospital data and to examine the distribution of cancer predisposition syndromes (CPSs) in a 20-year regional pediatric cohort. We present data collected between 2000 and 2019 from Karol Jonscher’s Clinical Hospital of Poznan University of Medical Sciences (KJCH PUMS), the region’s main pediatric oncology center, in relation to the pediatric population of the Greater Poland Region, using details provided by the Central Statistical Office of Poland. Given the increasing availability of genetic testing and advances in personalized therapies, we aim to determine CPS incidence from this data. To the best of our knowledge, no such comparative analysis has been published before.

## 2. Materials and Methods

The analyzed data had been collected over 20 years, between 2000 and 2019, from three institutions: Karol Jonsher’s Clinical Hospital of the Poznan University of Medical Sciences (KJCH PUMS), the Polish National Cancer Registry (PNCR), and the Central Statistical Office of Poland (CSO). Therefore, the study represents an unselected cohort of Caucasian children aged from birth up to the 18th year of age with newly diagnosed cancer in the background of the region’s pediatric population. We received approval from the PUMS Bioethics Committee for this research (Resolution No. KB-197/26).

Depending on the data source, the possibilities for obtaining data differed, as explained below.

### 2.1. Karol Jonsher’s Clinical Hospital of the Poznan University of Medical Sciences (KJCH PUMS)

A total of 2190 patients were included in our study, both male and female, aged from birth to the 18th year of age, who were newly diagnosed with any kind of neoplasm and categorized by ICD-10 codes at KJCH PUMS between 1 January 2000 and 31 December 2019. From this cohort, 193 (8.8%) patients had an underlying genetic disorder defined as a cancer predisposition syndrome (CPS), which is related to diagnosed neoplasms in the enrolled individuals included in our study. The CPS diagnoses were based on genetic testing (karyotype, NGS, WES, or WGS, depending on the exact case) and/or clinical symptoms (Supplementary Table S1).

The age is top–down limited because of the pediatric profile of our hospital—once an adolescent becomes of age, starting on the day of their 18th birthday, all diagnostic and therapeutic procedures take place in other medical centers intended for adults. This results in a lack of data for 18- and 19-year-olds and provides some context for the different age-group compartments analyzed in this study.

During the data collection process, patients were assigned to age groups using a one-month interval for infants up to 12 months of age and a one-year interval for older children.

### 2.2. The Polish National Cancer Registry (PNCR)

Aiming to present the most reliable data possible, we submitted an official request to the Polish National Cancer Registry (PNCR) to release its data. The data were intended to relate to the pediatric population of the Greater Poland Region, covering patients from our center by gender, age (in one-year intervals), and year of diagnosis. In response to our request, the data provided were aggregated. According to the Central Statistical Office of Poland (CSO) guidelines for public statistics, the Administrator of the PNCR was required to record zero cases for small subsets in the report prepared for us, resulting in a total of 230 patients, which appears to be significantly underreported. This compelled us to abandon analysis of the provided data due to the unrepresentative group, which was 9.5 times smaller than the cohort collected and treated at our center.

### 2.3. The Central Statistical Office of Poland (CSO)

Population data from the CSO are publicly accessible. All necessary reports, which form the background of the analyzed patient group, were generated through the official website: https://stat.gov.pl/ (accessed on 16 July 2024).

### 2.4. Statistical Software

All analyses were performed in Python 3.12 using the pandas (v2.3.1), numpy (v2.3.2), statsmodels (v0.14.5), scipy (v1.16.1), seaborn (v0.13.2), and matplotlib (v3.10.5) packages.

### 2.5. Statistical Analysis

Statistical methods were selected according to the aggregated age-stratified structure of the data and the expected sparsity of several CPS-related subgroups. Poisson and logistic regression models were used to estimate age-specific rates and associations, while non-parametric rank-based tests were applied to compare ordered age distributions without assuming normality. Chi-square goodness-of-fit tests were used to assess deviations between observed and expected age distributions. Exact tests or Monte Carlo procedures were preferentially used in sparse contingency tables to avoid reliance on asymptotic assumptions. False discovery rate (FDR) correction was applied across analyses to account for multiple testing.

#### 2.5.1. The Age-Specific Hospital-Based Case Rate of Childhood Cancer

Age-specific case counts were grouped into seven predefined age categories: (1) newborns up to 28 days old, (2) 1–2-month-olds, (3) 3–11-month-olds, (4) 1–2-year-olds, (5) 3–6-year-olds, (6) 7–15-year-olds, and (7) 16–17-year-olds. Population denominators were sourced from national statistics, with infants under 1 year divided into the three groups mentioned above, using proportional weights ($\frac{1}{12}$, $\frac{2}{12}$, and $\frac{9}{12}$ of the annual population, respectively).

Chi-square goodness-of-fit tests were conducted for each ICD-10 category to determine whether the observed age distribution differed from the expected distribution based on the population age structure. *p*-values were adjusted for multiple comparisons using the Benjamini–Hochberg false discovery rate (FDR, α = 0.05). Pearson residuals were calculated to identify the age groups that contributed most significantly to deviations from the expected distribution.

Hospital-based case rates across different age groups were estimated using Poisson regression models, with case counts as the dependent variable, age group as a categorical predictor, and log(population size) as an offset. For each ICD-10 code, the reference category was the youngest age group with at least five observed cases. Rate ratios (RR) with 95% confidence intervals were reported, and estimates based on fewer than five cases were marked as unstable and not discussed in the text.

Analyses were performed using 20-year aggregated data, and all tests were two-sided.

#### 2.5.2. Cancer Predisposition Syndromes (CPSs)

Age-specific hospital-based incidence rates (IR) per 100,000 person-years were calculated using exact Poisson 95% confidence intervals, based on population denominators from the Central Statistical Office of Poland (the Greater Poland Region, 2000–2019). Associations between age group and CPS positivity were evaluated using Poisson regression with log(person-years) as the offset. Logistic (binomial) regression was also used to model the odds of a CPS-positive cancer diagnosis, with age group as the predictor.

Age distributions across cancer predisposition syndromes (CPSs) were compared using a grouped permutation Kruskal–Wallis test on aggregated counts in the original ordered age strata, with Monte Carlo *p*-values. Post hoc pairwise comparisons were performed using grouped Wilcoxon–Mann–Whitney tests with Benjamini–Hochberg false discovery rate (FDR) correction. Only CPS categories with ≥3 cases were included.

Within each ICD-10 cancer category, age distributions of CPS-positive and CPS-negative patients were compared using grouped Wilcoxon–Mann–Whitney tests applied to aggregated counts in the original ordered age strata. Two-sided Monte Carlo *p*-values (50,000 iterations) and the common-language effect size A with bootstrap 95% confidence intervals were reported. *p*-values were adjusted using the Benjamini–Hochberg false discovery rate (FDR) procedure.

Additionally, differences in age-stratum distributions between CPS-positive and CPS-negative patients were assessed using 2 × k contingency tables with Fisher’s exact test (for 2 × 2 tables) or Monte Carlo chi-square tests (50,000 resamples; fixed marginal totals) for larger or sparse tables. Only ICD-10 categories with ≥20 total cases and ≥3 CPS-positive cases were included. All *p*-values were FDR-adjusted.

## 3. Results

### 3.1. Pediatric Population Background of the Greater Poland Region (2000–2019): The Central Statistical Office of Poland (CSO)

**Figure 1:**
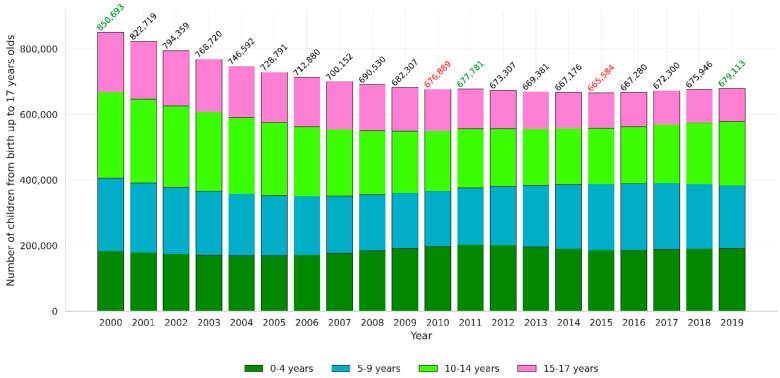
Number of children from birth up to 17 years old in the Greater Poland Region (2000–2019). Above the bars, the total number of children in the corresponding year is shown. The highest values are highlighted in green and the lowest in red, which illustrates the nonlinear nature of changes in the pediatric population. Data source: the Central Statistical Office of Poland.

According to Statistics Poland, the number of children aged 0–17 in the Greater Poland Region decreased from 850,693 in 2000 to 665,584 in 2015 (−21.7%), then increased modestly to 679,113 in 2019, as shown in Figure 1 for the years 2000–2019.

The age distribution of the pediatric population also shifted significantly: the percentage of children aged 0–4 years grew from 183,323 (21.6% of the total population) in 2000 to 196,885 (around 29%) in the early 2010s, then declined to 191,539 (28.2%) in 2019. Meanwhile, the proportion of adolescents aged 15–17 years steadily dropped from 182,893 (21.5%) in 2000 to 100,395 (14.8%) in 2019. As a result, the average age in the 0–17-year group decreased from 9.46 years in 2000 to about 8.3 years in 2017–2019.

### 3.2. Unselected Cohort of Caucasian Children Newly Diagnosed with Cancer Between 1 January 2000 and 31 December 2019: Karol Jonsher’s Clinical Hospital of the Poznan University of Medical Sciences (KJCH PUMS) Database

Over 20 years of data collection, the cohort included 2190 children from birth up to 17 years old with newly diagnosed neoplasms. Males predominated among our patients, accounting for 1233 cases (56.3%), compared to 957 females (43.7%). The average age at diagnosis in our cohort was 7.5 years (SD ± 5.5). The most common diagnoses can be grouped into three main categories: (1) leukemias, represented by ICD-10 codes C91, C92, C94, and C95, with 704 patients (32.1%), (2) central nervous system (CNS) tumors, with ICD-10 codes C70–C72, comprising 382 children (17.4%), and (3) lymphomas, classified under ICD-10 codes C81–C85, totaling 279 patients (12.7%). Together, these groups account for 1353 cases (61.8%), including both males and females.

**Figure 2:**
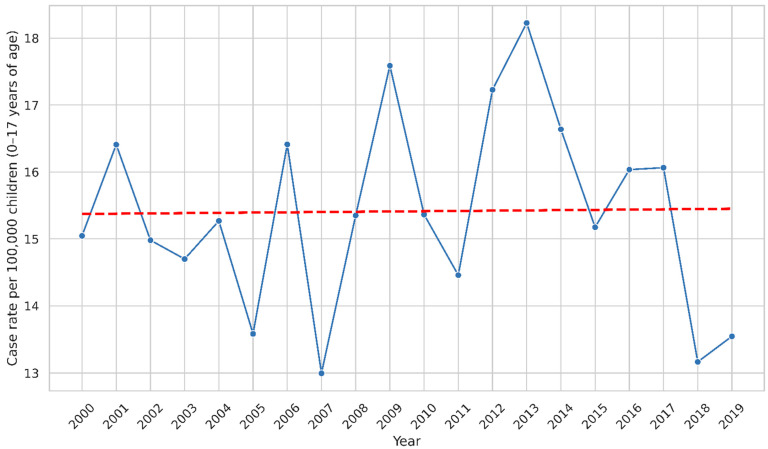
Annual childhood cancer hospital-based case rates per 100,000 children (0–17 years of age) show a slightly upward trend (dashed line); however, this trend is not statistically significant (RR per year: 1.00, 95% CI 0.99–1.01, *p* = 0.94). KJCH PUMS case rates reflect the number of cases diagnosed at a single tertiary center relative to the regional population and should not be interpreted as population incidence.

Annual new case numbers ranged from 89 to 135 patients. The data corresponded to the pediatric population between 2000 and 2019, allowing the calculation of annual childhood cancer hospital-based case rates per 100,000 children, ranging from 13.0 in 2007 to 18.2 in 2013 (Figure 2).

**Figure 3:**
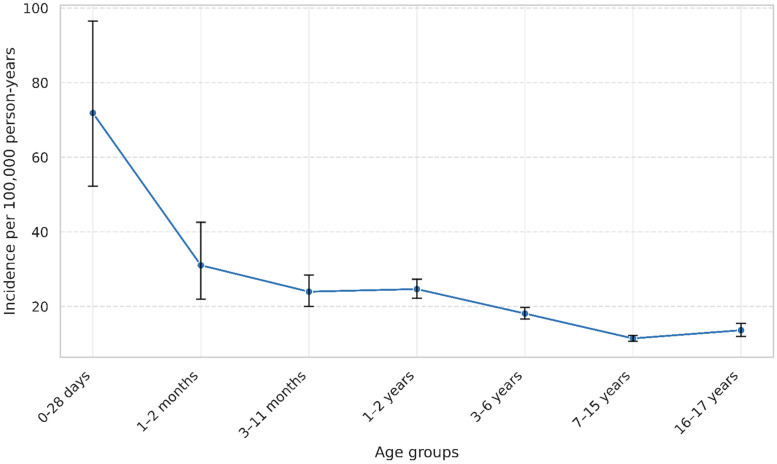
Age-specific hospital-based case rates of childhood cancer per 100,000 person-years. Error bars represent 95% Poisson confidence intervals.

The age-specific hospital-based case rate of childhood cancer (all types combined) peaked in the 0–28 days age group at 71.8 per 100,000 person-years (95% CI: 52.2–96.4). Incidence rates dropped sharply during infancy and early childhood, reaching 11.4 per 100,000 (95% CI: 10.7–12.3) in the 7–15 years group, with a slight increase observed among adolescents aged 16–17 years (13.6 per 100,000, 95% CI: 12.0–15.4; Figure 3).

Poisson regression showed a declining risk with age, decreasing from about 2-fold to 6-fold lower than the 0–28 days reference group (RR = 0.16–0.43, all *p* < 0.001; Table 1).

**Figure 4:**
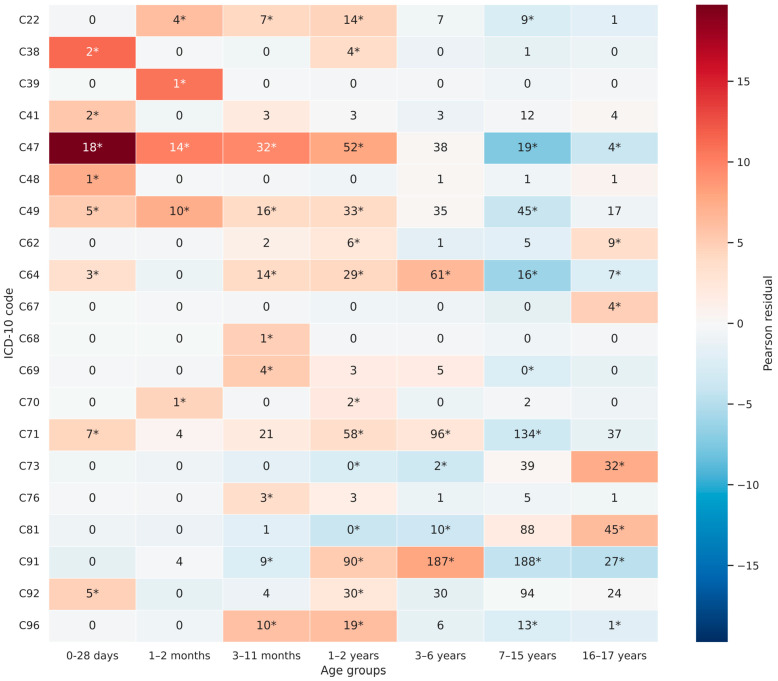
Age distribution of childhood cancer cases by age groups for ICD-10 categories with significant deviations from the population’s age structure (χ^2^ goodness-of-fit test, *P*_FDR_ < 0.05). Colors show Pearson residuals indicating deviations from the expected population-based distribution (red: more cases than expected; blue: fewer). Numbers indicate observed case counts. Asterisks (*) mark cells with |Pearson residual| > 1.96. For ICD-10 categories with very small case numbers, results should be interpreted with caution.

Age distributions differed significantly from the population age structure in twenty of thirty-seven ICD-10 categories after Benjamini–Hochberg correction (χ^2^ goodness-of-fit test, *P*_FDR_ < 0.05) (Figure 4; Supplementary Table S2). Inspection of Pearson residuals revealed distinct age-specific patterns across tumor types. Several categories showed strong overrepresentation in the youngest age groups, including liver tumors (C22), mediastinal tumors (C38), and peripheral nerve and autonomic nervous system tumors (C47). In contrast, other malignancies were more common in older children and adolescents, including thyroid cancer (C73) and Hodgkin lymphoma (C81). Some extreme residual values in the youngest age groups were partly driven by very small, expected counts and should therefore be interpreted cautiously.

Age-specific rate ratios (RRs) were calculated using Poisson regression models with the log of the population size as an offset. For each ICD-10 category, the reference group was automatically chosen as the youngest age group with at least five observed cases. Only estimates based on five or more cases were considered. Notable findings include:

1. Malignant neoplasm of the liver and intrahepatic bile ducts (C22): Lower rate ratios were seen in older age groups, with RR = 0.098 (95% CI: 0.037–0.264) in the 7–15-year-olds compared to the 3–11-month-olds reference group.
2. Malignant neoplasm of peripheral nerves and the autonomic nervous system (C47): Rate ratios decreased steadily across age groups, reaching RR = 0.009 (95% CI: 0.005–0.017) in 7–15-year-olds compared to the 0–28-day-old reference group.
3. Malignant neoplasm of soft tissue (C49): Compared to the reference group of 0–28-day-olds, lower rate ratios were seen in all subsequent age groups, with RR = 0.076 (95% CI: 0.030–0.192) in 7–15-year-olds and RR = 0.115 (95% CI: 0.042–0.311) in 16–17-year-olds.
4. Malignant neoplasm of the kidney (C64): Rate ratios decreased in older age groups, with RR = 0.087 (95% CI: 0.043–0.179) in the 7–15-year-olds compared to the 3–11-month-olds reference group.
5. Malignant neoplasm of the brain (C71): A decreasing trend in rate ratios was observed with increasing age, with RR = 0.162 (95% CI: 0.076–0.347) in 7–15-year-olds compared to the 0–28-day-old reference group.
6. Hodgkin lymphoma (C81): Higher rate ratios were seen in older age groups, with RR = 7.39 (95% CI: 3.72–14.66) in the 16–17-year-olds compared to the 3–6-year-olds reference group.
7. Lymphoid leukemia (C91): Higher rate ratios were seen in the 1–2-year-old group (RR = 3.72, 95% CI: 1.88–7.38) and the 3–6-year-old group (RR = 3.85, 95% CI: 1.97–7.52) compared to the 3–11-month-olds reference group.
8. Myeloid leukemia (C92): Lower rate ratios were seen in older age groups, with RR = 0.124 (95% CI: 0.048–0.318) in the 3–6-year-olds compared to the 0–28-day-olds reference group.

Detailed age-specific RR estimates with 95% confidence intervals for all ICD-10 categories are available in Supplementary Table S3.

### 3.3. Cancer Predisposition Syndromes (CPSs) as an Underlying Cause of Oncological Diagnosis Among Pediatric Patients’ Cohort

Out of 2190 children diagnosed with cancer at our center over 20 years, 193 (8.8%) had a confirmed cancer predisposition syndrome (CPS), either pre-existing or identified subsequently, that matched their oncological disease. The highest proportion of CPS-positive cases was observed in infancy and gradually decreased with age (Table 2). The age-specific incidence of CPS-positive cancers dropped from 18.0 (95% CI: 8.2–29.4) per 100,000 person-years in the first month of life to 0.7 (95% CI: 0.3–1.2) in 16–17-year-olds.

Poisson regression confirmed a strong inverse relationship with age. Compared to the first month of life, rate ratios decreased progressively across older age groups, from 0.41 (95% CI: 0.17–0.99) in 1–2-month-olds to 0.04 (95% CI: 0.02–0.09) in 7–15-year-olds. Binomial regression estimating the odds of CPS-positive cancer diagnosis showed a similar age-related pattern.

**Figure 5:**
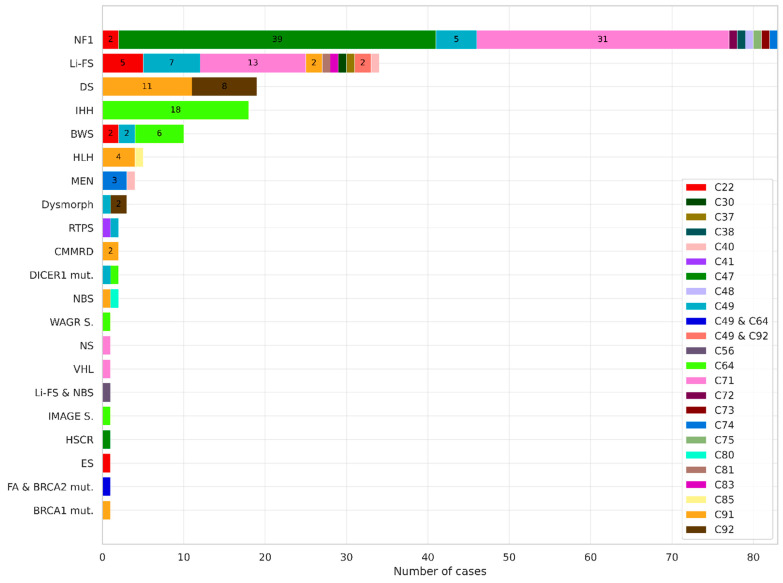
Cancer occurrence (presented with ICD-10 codes) among CPS-positive patients in the cohort. BWS: Beckwith–Wiedemann Syndrome, CMMRD: Constitutional Mismatch Repair Deficiency Syndrome, DS: Down Syndrome, Dysmorph: dysmorphic features, ES: Edwards’ Syndrome, FA: Fanconi anemia, HSCR: Hirschsprung disease, HLH: Hemophagocytic lymphohistiocytosis, IHH: Isolated hemihypertrophy, Li–FS: Li–Fraumeni Syndrome, MEN: Multiple endocrine neoplasia Syndrome, mut.: gene mutation, NBS: Nijmegen Breakage Syndrome, NF1: Neurofibromatosis type I, NS: Noonan Syndrome, RTPS: Rhabdoid Tumor Predisposition Syndrome, S.: Syndrome, VHL: Von Hippel–Lindau Syndrome, and (&): co-occurrence; ICD-10 codes: C22: Malignant neoplasm of liver and intrahepatic bile ducts, C30: Malignant neoplasm of the nasal cavity and middle ear, C37: Malignant neoplasm of thymus, C38: Malignant neoplasm of the heart, mediastinum and pleura, C40: Malignant neoplasm of bone and articular cartilage of limbs, C41: Malignant neoplasm of bone and articular cartilage of other and unspecified sites, C47: Malignant neoplasm of peripheral nerves and autonomic nervous system, C49: Malignant neoplasm of other connective and soft tissue, C56: Malignant neoplasm of ovary, C64: Malignant neoplasm of kidney, except renal pelvis, C71: Malignant neoplasm of the brain, C72: Malignant neoplasm of the spinal cord, cranial nerves and other parts of the central nervous system, C73: Malignant neoplasm of the thyroid gland, C74: Malignant neoplasm of the adrenal gland, C75: Malignant neoplasm of other endocrine glands and related structures, C80: Malignant neoplasm, without specification of the site, C81: Hodgkin lymphoma, C83: Non-follicular lymphoma, C85: Other and unspecified types of non-Hodgkin lymphoma, C91: Lymphoid leukemia, C92: Myeloid leukemia.

The occurrence and distribution of cancers among CPS-positive patients are shown in Figure 5. Neurofibromatosis type 1 (NF1; n = 83, 43.0%), Li–Fraumeni Syndrome (Li-FS; n = 34, 17.6%), Down Syndrome (DS; n = 19, 9.8%), Isolated hemihypertrophy (IHH; n = 18, 9.3%), and Beckwith–Wiedemann Syndrome (BWS; n = 10, 5.2%) together account for about 85% of all CPS-positive cases. An additional 29 patients had conditions including Hemophagocytic lymphohistiocytosis (HLH), Multiple Endocrine Neoplasia Syndrome (MEN), dysmorphic features, Constitutional Mismatch Repair Deficiency Syndrome (CMMRD), Nijmegen Breakage Syndrome (NBS), DICER1 mutation, Rhabdoid Tumor Predisposition Syndrome (RTPS), with at least two cases noted in each. The remaining CPSs, such as BRCA1 mutation, IMAGE Syndrome, Hirschsprung disease, Von Hippel–Lindau Syndrome (VHL), Edwards’ Syndrome (ES), Noonan Syndrome (NS), or WAGR Syndrome, were less common in our cohort. In two cases, the co-occurrence of two different CPSs was observed: Li–FS with NBS in a girl with ovarian cancer, and Fanconi Anemia (FA) with a BRCA2 mutation in a girl diagnosed with both rhabdomyosarcoma (RMS) and nephroblastoma.

**Figure 6:**
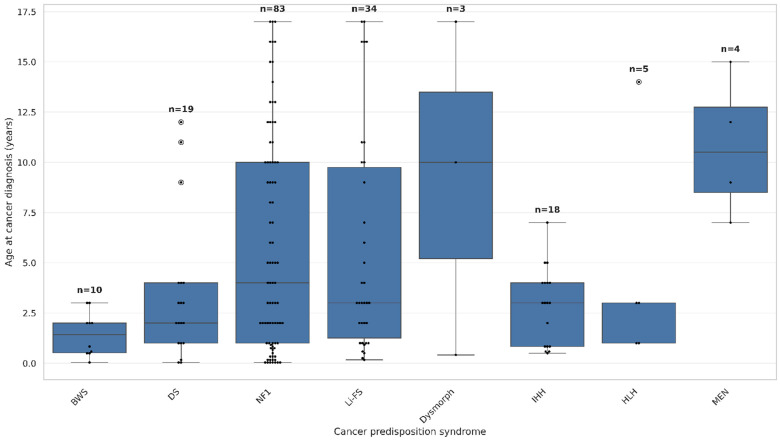
Age at neoplasm diagnosis by cancer predisposition syndrome (CPS) category (syndromes with three or more cases). BWS: Beckwith–Wiedemann Syndrome, DS: Down Syndrome, Dysmorph: dysmorphic features, HLH: Hemophagocytic lymphohistiocytosis, IHH: Isolated hemihypertrophy, Li-FS: Li–Fraumeni Syndrome, MEN: Multiple Endocrine Neoplasia Syndrome, NF1: Neurofibromatosis type I.

Age at neoplasm diagnosis differed significantly across cancer predisposition syndromes (CPSs) (grouped permutation Kruskal–Wallis test: H = 14.63, *p* = 0.033; Figure 6). Post hoc pairwise comparisons with FDR correction showed that MEN patients were diagnosed at a significantly older age than those with BWS (adjusted *p* = 0.028), IHH (adjusted *p* = 0.006), and DS (adjusted *p* = 0.042). No other pairwise comparisons were statistically significant.

Within each ICD-10 cancer category, CPS-positive patients were diagnosed at a significantly younger age than CPS-negative patients, as determined by the grouped Wilcoxon–Mann–Whitney test on the original ordered age strata. Significant differences were observed for liver and intrahepatic bile duct tumors (C22: A = 0.097, adjusted *p* < 0.001), myeloid leukemia (C92: A = 0.179, adjusted *p* < 0.001), lymphoid leukemia (C91: A = 0.309, adjusted *p* = 0.007), and renal tumors (C64: A = 0.335, adjusted *p* = 0.013). No significant age differences were found for soft-tissue sarcomas (C49), brain tumors (C71), or peripheral nerve and autonomic nervous system tumors (C47) after FDR correction (all adjusted *p* ≥ 0.300) (Table 3).

Contingency table analyses identified a significant difference in age-stratum distribution for liver tumors (C22: Monte Carlo χ^2^ = 32.69, adjusted *p* = 0.010). Borderline differences were observed for renal tumors (C64), peripheral nerve and autonomic nervous system tumors (C47), soft-tissue sarcomas (C49), and myeloid leukemia (C92), but none reached statistical significance after FDR correction (all adjusted *p* ≈ 0.051).

## 4. Discussion

As far as we know, this is the first study summarizing the incidence of pediatric neoplasms among children with cancer predisposition syndromes (CPSs) in a regional population aged from birth to 17 years, using 20 years of data collected between 2000 and 2019 in Poland.

The data obtained from the Polish National Cancer Registry (PNCR), due to their unrepresentative nature and our precise request (described in the Materials and Methods section), along with the Central Statistical Office of Poland (CSO) guidelines for public statistics requirements, were excluded from the statistical analysis. As the PNCR Administrator noted, the indicated age ranges consistently contained small sample sizes (under five cases). In accordance with the CSO guidelines, the Administrator was forced to enter a value of zero under such conditions. We were warned that expanding the age intervals to five-year age groups would still result in missing cases in the summary, as long as they were not reported as individual records. Even without the age intervals, the Administrator had to apply the described scheme for data display to fewer than five records. This resulted in the PNCR providing 230 cases, without our request changing, compared to 2190 patients diagnosed and treated at our center, showing a discrepancy of over 9.5-fold. Omitting this analysis remains the biggest limitation of this study. Other limitations include the single-center database and hospital-based rates. However, these may be justified by the Pediatric Oncology and Hematology Clinic’s central location in the Greater Poland Region and its status as the only facility of its kind in the entire voivodeship. Nevertheless, the observed trends cannot be interpreted as population incidence since the data come from a single center with a high likelihood of incompleteness, partly due to medical tourism and patients migrating to closer centers (for residents near the voivodeship borders) or specialized centers for rare cases (such as retinoblastoma, where the reference institute is located in Warsaw).

The medical tourism phenomenon presents some interpretive challenges. First, we lack sufficient data to precisely determine its regional scope—that was not the aim of our study. However, we observed patient migration between Polish provinces, especially at the beginning of the twenty-first century, when pediatric oncology departments were not available in every region. In cases of necessity, treatment was complemented with hematopoietic stem cell transplantation (HSCT): our hospital offers this treatment for children as one of only six such centers in the country to date. We are also aware of personal cases where families decided to pursue therapy in countries like Germany, Italy, Spain, or the USA after exhausting the options available in Poland. These decisions are often driven by differences in the registration and availability of specific medications, especially when the prognosis worsens or standard therapy fails to produce the expected results.

A slight increasing trend of annual childhood cancer case rates observed among our cohort occurred but was not statistically significant (RR per year 1.00, 95% CI 0.99–1.01, *p* = 0.94) (Figure 2). What is interesting, however, is that the newest official national report summarizing cancer incidence in the whole population of Poland [17], as well as researchers studying pediatric oncology patients up to 17 years of age across the country [32], confirm this finding. Although the number of children newly diagnosed with neoplasms has remained relatively constant in Poland for over 20 years, as J. R. Kowalczyk et al. present in their paper, the Polish pediatric population is decreasing, which may explain the observed rising trend in cancer incidence. Also, global childhood cancer incidence trends seem to be increasing [3,33,34,35,36,37,38]. This may be the result of diagnostic testing and imaging development, the establishment of algorithms, and the availability of special procedures in general.

Analysis of age-specific rate ratios (RR) for ICD-10 codes (see “stable” estimates described in the Results section) shows an infancy predominance of neoplasms of embryonal origin, such as hepatoblastoma, neuroblastoma, and nephroblastoma. A meaningful difference in age dispersion at neoplasm diagnosis with an underlying CPS occurred for patients with C22 tumors: CPS-positive and CPS-negative children were unevenly distributed among age categories at the time of cancer detection. What is interesting, children with an underlying CPS were diagnosed with leukemia (both lymphoid and myeloid), C22 and C64 tumors (most commonly hepatoblastoma and nephroblastoma, (Wilms tumor)), significantly earlier than CPS-negative patients with identical oncological diagnosis (Table 3). Our observation is corroborated by the literature and underscores the need for surveillance of CPS-positive patients [39,40,41,42]. Also, among children with any kind of blastoma cancer, CPS co-occurred in over $\frac{1}{5}$ of cases in each corresponding group of the ICD-10 classification (20.8% of patients with C64 tumors, 22.6% of patients with C47 tumors, and 23.8% of patients with C22 tumors, respectively), resulting in 77 CPS-positive patients (39.9% of all cases with an underlying CPS in our cohort) representing the most representative group, followed by 46 CPS-positive patients with C71 tumors (including medulloblastoma, another kind of blastoma cancer). Researchers have proposed a model of embryonal tumorigenesis, concerning childhood cancers with clinical evidence of a prenatal origin (including two types of solid tumors: neuroblastoma and medulloblastoma, and two types of hematopoietic malignancies: myeloid leukemia–Down syndrome and B-lineage acute lymphoblastic leukemia). Their common features include: (1) proliferative excess in the prenatal period affecting the origin tissue, (2) an intracellular mechanism of death resistance and persistence of precancerous cells in a challenging postnatal environment, and (3) a genomic instability pathway [43]. This corresponds to Knudson’s earlier two-hit thesis, proposed after a brief observation of 48 children diagnosed with retinoblastoma, which states that the second mutation is necessary for carcinogenesis and occurs after birth, while the first is more often inherited. In cases of such congenital genetic predisposition, the tumor was detected significantly earlier compared to children with both postnatal mutations [44]. Within a year, he expanded his theory to other cancers: neuroblastoma, pheochromocytoma, and nephroblastoma [45,46]. However, after a brief literature overview, we could not find any study related to our observation that combines all or most types of malignancies arising from precursor cells (blastomas) and their co-occurrence with underlying CPSs. This observation requires further investigation and may provide the basis for crucial discoveries in pediatric medicine.

The prevalence of CPSs among oncological patients in our pediatric population is likely underestimated. In the studied cohort, 193 of 2190 children had known CPSs that could be diagnosed with available tests, representing 8.8% of all cases, while among patients with central nervous system (CNS) tumors, 12.9% were diagnosed with an underlying CPS. This corresponds to worldwide tendencies, suggesting that the CPS incidence ranges between 8.5% and 18% among children with cancer in general, and up to 21% among pediatric patients with CNS tumors [11,47,48,49,50,51]. The awareness of a potential cause of cancer in a child, which is not typical or common in itself, should lead specialists to refer the patient (sometimes together with family members) to genetic counseling, testing, and individualized vigilant surveillance [2]. This creates two significant areas for action—financial, to fund all additional examinations, and educational, including general practitioners, pediatricians, neonatologists, radiologists, and parents of affected children. In Poland, unfortunately, the healthcare and medical sector is underfunded, which results in the necessity of raising funds for recommended but unaffordable tests from foundations or covering the costs personally for patients and their families. This is the first limitation of specialized genetic testing for CPSs detected in our cohort. The second limitation is time—over the last decades, several fields of medicine have improved significantly and have become more accessible: genetic testing, including the next generation sequencing (NGS) and whole exome or genome sequencing (WES, WGS), as well as advances in engineering, diagnostic strategies, biological treatment, immunotherapy, and others, providing the possibility not only to detect CPSs, but also to preform risk stratification at the time of diagnosis and deliver personalized therapy adequate to the underlying genetic findings [9,52,53,54,55]. The benefits of treatment and surveillance adjustment are invaluable: better prognosis and therapy response, reduced side effects and toxicity (including, e.g., radiation) related to underlying CPS, and vigilant follow-up enabling early detection of secondary carcinogenesis [56,57,58]. Therefore, many research groups have undertaken efforts to establish guidelines worth following for several CPSs [39,47,59,60,61,62,63,64,65,66,67,68,69,70,71,72]. Unfortunately, a neoplasm is often the first manifestation of a CPS. In such cases, the cancer can likely reach an advanced stage with an unfavorable prognosis. The prior diagnosis of a CPS leading to primary rather than secondary cancer prevention or early detection remains challenging. Proposed questionnaires intended to select patients for genetic referral and CPS confirmation, including the MIPOGG and Jongmans et al. criteria, have not been fully effective so far, leading to missed detection of genetic disorders and inadequate surveillance, suggesting that CPS diagnostics should be undertaken much more frequently than recommended [73,74,75,76,77,78,79]. All medical professionals combined should encourage ministry authorities to recognize that prevention carries lower costs, both financial for the government and psychological for the individual, and brings many more benefits than necessary and aggressive treatment thereafter.

## 5. Conclusions

The pediatric population in the Greater Poland Region has been declining over the two decades of our observation. Although there has been a slight increase in the hospital-based cancer incidence trend, this change was not statistically significant. However, the national report, which includes the period of our data collection, confirms this trend against the backdrop of a decreasing number of children in Poland overall. Cancer predisposition syndromes (CPSs) are important and likely underestimated contributors to pediatric malignancies, especially in early childhood. The earlier onset of cancer among CPS-positive patients highlights the need for systematic genetic assessment and surveillance. Expanding access to genetic diagnostics may significantly improve risk stratification, personalized therapy, and long-term outcomes in pediatric oncology.

## Figures and Tables

**Table 1:** Poisson regression results for overall childhood cancer hospital-based case rates by age group. Reference: 0–28-day-olds. *p* less than 0.001 are summarized with three asterisks (***).

Age Group	coef	RR	95% CI	*p*
1–2-month-olds	−0.8398	0.432	0.280–0.667	<0.001 ***
3–11-month-olds	−1.0986	0.333	0.237–0.469	<0.001 ***
1–2-year-olds	−1.4749	0.229	0.167–0.313	<0.001 ***
3–6-year-olds	−1.3860	0.250	0.184–0.340	<0.001 ***
7–15-year-olds	−1.7260	0.178	0.131–0.241	<0.001 ***
16–17-year-olds	−1.6624	0.190	0.138–0.261	<0.001 ***

**Table 2:** Distribution and age-specific hospital case rates of CPS-positive patients, expressed per 100,000 person-years (exact Poisson 95% CI), based on 20-year accumulated person-years for each age group. CPS(+): CPS-positive.

Age Group	Number of Patients		Incidence per 100,000 Person-Years	95% CI
	with Cancer	CPS(+) (%)		
0–28-day-olds	44	11 (25.0)	18.0	8.2–29.4
1–2-month-olds	38	9 (23.7)	7.3	3.3–12.2
3–11-month-olds	132	29 (22.0)	5.3	3.4–7.3
1–2-year-olds	365	37 (10.1)	1.7	1.2–2.2
3–6-year-olds	538	51 (9.5)	1.7	1.3–2.2
7–15-year-olds	826	43 (5.2)	0.7	0.5–0.9
16–17-year-olds	247	13 (5.3)	0.7	0.3–1.2

**Table 3:** Comparison of age-at-diagnosis distributions between CPS-positive and CPS-negative patients by ICD-10 cancer type (grouped Wilcoxon–Mann–Whitney test on ordered age strata; Benjamini–Hochberg FDR correction). Median age band indicates the stratum containing the 50th percentile of cases. A denotes the common-language effect size. Adj. *p*: adjusted *p*, ANS: autonomic nervous system, CPS(+): CPS-positive, CPS(−): CPS-negative, M. neo.: malignant neoplasm, m. of l.: month of life, y. of l.: year of life. Adj. *p* less than 0.001 are summarized with three asterisks (***), less than 0.01 with two asterisks (**), and less than 0.05 with one asterisk (*) only.

ICD-10	Cancer Type	Number of Cases		Median Age Band		A (95% CI)	Adj. *p*
		CPS(+)	CPS(−)	CPS(+)	CPS(−)		
C22	M. neo. of the liver and intrahepatic bile ducts	10	32	6th m. of l.	3rd y. of l.	0.097 (0.000–0.239)	<0.001 ***
C92	Myeloid leukemia	10	177	2nd y. of l.	11th y. of l.	0.179 (0.069–0.311)	<0.001 ***
C91	Lymphoid leukemia	21	484	4th y. of l.	6th y. of l.	0.309 (0.202–0.427)	0.007 **
C64	M. neo. of the kidney, except renal pelvis	27	103	4th y. of l.	5th y. of l.	0.335 (0.233–0.446)	0.013 *
C49	M. neo. of other connective and soft tissue	17	144	3rd y. of l.	5th y. of l.	0.408 (0.269–0.556)	0.304
C71	M. neo. of the brain	46	311	6th y. of l.	7th y. of l.	0.456 (0.371–0.542)	0.395
C47	M. neo. of the peripheral nerves and the ANS	40	137	3rd y. of l.	2nd y. of l.	0.544 (0.424–0.659)	0.398

## Data Availability

The data presented in this study are available in the Supplementary Material.

## Supplementary Materials

The following supporting information can be downloaded at: https://www.mdpi.com/article/10.3390/children13060778/s1, Supplementary Table S1: The list of all cancer predisposition syndromes (CPSs) and related clinical features observed among the group of 193 CPS-positive patients; Supplementary Table S2: Chi-square goodness-of-fit test results comparing observed and expected age distributions across ICD-10 categories; Supplementary Table S3: Age-specific RR estimates with 95% confidence intervals for all ICD-10 categories.

## References

[1] Siegel R.L., Giaquinto A.N., Jemal A. (2024). Cancer Statistics, 2024. CA Cancer J. Clin..

[2] Kentsis A. (2020). Why Do Young People Get Cancer?. Pediatr. Blood Cancer.

[3] Steliarova-Foucher E., Colombet M., Ries L.A.G., Moreno F., Dolya A., Bray F., Hesseling P., Shin H.Y., Stiller C.A. (2017). IICC-3 contributors. International Incidence of Childhood Cancer, 2001–10: A Population-Based Registry Study. Lancet Oncol..

[4] Rodriguez R., Krishnan Y. (2023). The Chemistry of Next-Generation Sequencing. Nat. Biotechnol..

[5] Shahani S.A., Marcotte E.L. (2022). Landscape of Germline Cancer Predisposition Mutations Testing and Management in Pediatrics: Implications for Research and Clinical Care. Front. Pediatr..

[6] Newman S., Nakitandwe J., Kesserwan C.A., Azzato E.M., Wheeler D.A., Rusch M., Shurtleff S., Hedges D.J., Hamilton K.V., Foy S.G. (2021). Genomes for Kids: The Scope of Pathogenic Mutations in Pediatric Cancer Revealed by Comprehensive DNA and RNA Sequencing. Cancer Discov..

[7] Benton M.L., Abraham A., LaBella A.L., Abbot P., Rokas A., Capra J.A. (2021). The Influence of Evolutionary History on Human Health and Disease. Nat. Rev. Genet..

[8] Sąsiadek M.M., Łaczmańska I., Maciejczyk A., Matkowski R., Gil J. (2020). Genetyka i onkologia (część 1.). Podstawy medycyny personalizowanej w onkologii opartej na badaniach genetycznych. Biul. Pol. Tow. Onkol. Nowotw..

[9] Nakagawa H., Fujita M. (2018). Whole Genome Sequencing Analysis for Cancer Genomics and Precision Medicine. Cancer Sci..

[10] Sabour L., Sabour M., Ghorbian S. (2017). Clinical Applications of Next-Generation Sequencing in Cancer Diagnosis. Pathol. Oncol. Res..

[11] Zhang J., Walsh M.F., Wu G., Edmonson M.N., Gruber T.A., Easton J., Hedges D., Ma X., Zhou X., Yergeau D.A. (2015). Germline Mutations in Predisposition Genes in Pediatric Cancer. N. Engl. J. Med..

[12] Nofi C.P., Roberts B.K., Rich B.S., Glick R.D. (2024). Pediatric, Adolescent and Young Adult (AYA) Peritoneal and Pleural Mesothelioma: A National Cancer Database Review. J. Pediatr. Surg..

[13] Bitner B.F., Lehrich B.M., Abiri A., Yasaka T.M., Hsu F.P.K., Kuan E.C. (2021). Characteristics and Overall Survival in Pediatric versus Adult Pituitary Adenoma: A National Cancer Database Analysis. Pituitary.

[14] Vujanić G.M., Gessler M., Ooms A.H.A.G., Collini P., Coulomb-l’Hermine A., D’Hooghe E., de Krijger R.R., Perotti D., Pritchard-Jones K., Vokuhl C. (2018). The UMBRELLA SIOP-RTSG 2016 Wilms Tumour Pathology and Molecular Biology Protocol. Nat. Rev. Urol..

[15] Maizlin I.I., Nice T.R., Onwubiko C., Goldfarb M., Gow K.W., Langer M., Doski J.J., Goldin A., Beierle E.A. (2018). Pediatric Patients with Small-Cell Carcinoma of the Ovary Have Similar Survival to Adults: A Review of the National Cancer Database. Am. Surg..

[16] Liu M., Thakkar J.P., Garcia C.R., Dolecek T.A., Wagner L.M., Dressler E.V.M., Villano J.L. (2018). National Cancer Database Analysis of Outcomes in Pediatric Glioblastoma. Cancer Med..

[17] Didkowska J., Barańska K., Miklewska M.J., Wojciechowska U. (2024). Cancer Incidence and Mortality in Poland in 2023. Nowotw. J. Oncol..

[18] Wojtyła C., Bertuccio P., Giermaziak W., Santucci C., Odone A., Ciebiera M., Negri E., Wojtyła A., La Vecchia C. (2023). European Trends in Ovarian Cancer Mortality, 1990–2020 and Predictions to 2025. Eur. J. Cancer.

[19] Ward Z.J., Yeh J.M., Bhakta N., Frazier A.L., Atun R. (2019). Estimating the Total Incidence of Global Childhood Cancer: A Simulation-Based Analysis. Lancet Oncol..

[20] Mullen C.J.R., Barr R.D., Franco E.L. (2021). Timeliness of Diagnosis and Treatment: The Challenge of Childhood Cancers. Br. J. Cancer.

[21] Lupo P.J., Spector L.G. (2020). Cancer Progress and Priorities: Childhood Cancer. Cancer Epidemiol. Biomark. Prev..

[22] Fair D., Potter S.L., Venkatramani R. (2020). Challenges and Solutions to the Study of Rare Childhood Tumors. Curr. Opin. Pediatr..

[23] Ferrari A., Brecht I.B., Gatta G., Schneider D.T., Orbach D., Cecchetto G., Godzinski J., Reguerre Y., Bien E., Stachowicz-Stencel T. (2019). Defining and Listing Very Rare Cancers of Paediatric Age: Consensus of the Joint Action on Rare Cancers in Cooperation with the European Cooperative Study Group for Pediatric Rare Tumors. Eur. J. Cancer.

[24] Gatta G., Trama A., Capocaccia R., RARECARENet Working Group (2019). Epidemiology of Rare Cancers and Inequalities in Oncologic Outcomes. Eur. J. Surg. Oncol..

[25] Pappo A.S., Furman W.L., Schultz K.A., Ferrari A., Helman L., Krailo M.D. (2015). Rare Tumors in Children: Progress Through Collaboration. J. Clin. Oncol..

[26] Wu Z., Xia F., Wang W., Zhang K., Fan M., Lin R. (2024). Worldwide Burden of Liver Cancer across Childhood and Adolescence, 2000–2021: A Systematic Analysis of the Global Burden of Disease Study 2021. EClinicalMedicine.

[27] Liu K., Shao J., Cai J., Tang J., Shen S., Xu F., Ren Y., Zhang A., Tian X., Lu X. (2023). Causes of Death and Treatment-Related Mortality in Newly Diagnosed Childhood Acute Lymphoblastic Leukemia Treatment with Chinese Children’s Cancer Group Study ALL-2015. Ann. Hematol..

[28] Becker C., Graf N., Grabow D., Creutzig U., Reinhardt D., Weyer-Elberich V., Spix C., Kaatsch P. (2020). Early Deaths from Childhood Cancer in Germany 1980–2016. Cancer Epidemiol..

[29] Suh E., Stratton K.L., Leisenring W.M., Nathan P.C., Ford J.S., Freyer D.R., McNeer J.L., Stock W., Stovall M., Krull K.R. (2020). Late Mortality and Chronic Health Conditions in Long-Term Survivors of Early-Adolescent and Young Adult Cancers: A Retrospective Cohort Analysis from the Childhood Cancer Survivor Study. Lancet Oncol..

[30] Peterson J., Wilson T.F., Watt M.H., Gruhl J., Davis S., Olsen J., Parsons M.W., Kann B.H., Swire-Thompson B., Fagerlin A. (2023). International Medical Tourism of US Cancer Patients for Alternative Cancer Treatments: Financial, Demographic, and Clinical Profiles of Online Crowdfunding Campaigns. Cancer Med..

[31] Zhong L., Deng B., Morrison A.M., Coca-Stefaniak J.A., Yang L. (2021). Medical, Health and Wellness Tourism Research-A Review of the Literature (1970–2020) and Research Agenda. Int. J. Environ. Res. Public Health.

[32] Kowalczyk J.R., Dudkiewicz E., Dembowska-Bagińska B., Balwierz W., Kazanowska B., Hetman M., Romiszewski M., Smalisz K., Książek A., Wachowiak J. (2025). Increasing Childhood Cancer Incidence in Poland in 1996–2020. Eur. J. Cancer.

[33] Kong Y., Ji X., Han X., Zhang B. (2022). Pediatric Neurological Cancer Incidence and Trends in the United States, 2000–2018. Cancer Causes Control.

[34] Grabas M.R., Kjaer S.K., Frederiksen M.H., Winther J.F., Erdmann F., Dehlendorff C., Hargreave M. (2020). Incidence and Time Trends of Childhood Cancer in Denmark, 1943–2014. Acta Oncol..

[35] Paapsi K., Baburin A., Mikkel S., Mägi M., Saks K., Innos K. (2020). Childhood Cancer Incidence and Survival Trends in Estonia (1970–2016): A Nationwide Population-Based Study. BMC Cancer.

[36] Bernier M.-O., Withrow D.R., Berrington de Gonzalez A., Lam C.J.K., Linet M.S., Kitahara C.M., Shiels M.S. (2019). Trends in Pediatric Thyroid Cancer Incidence in the United States, 1998–2013. Cancer.

[37] Schwartz I., Hughes C., Brigger M.T. (2015). Pediatric Head and Neck Malignancies: Incidence and Trends, 1973–2010. Otolaryngol. Head. Neck Surg..

[38] Hosny G., Elkaffas S.M. (2002). Patterns in the Incidence of Pediatric Cancer in Alexandria, Egypt, from 1972 to 2001. J. Egypt. Public Health Assoc..

[39] Kalish J.M., Becktell K.D., Bougeard G., Brodeur G.M., Diller L.R., Doria A.S., Hansford J.R., Klein S.D., Kohlmann W.K., Kratz C.P. (2024). Update on Surveillance for Wilms Tumor and Hepatoblastoma in Beckwith-Wiedemann Syndrome and Other Predisposition Syndromes. Clin. Cancer Res..

[40] Mussa A., Duffy K.A., Carli D., Griff J.R., Fagiano R., Kupa J., Brodeur G.M., Ferrero G.B., Kalish J.M. (2019). The Effectiveness of Wilms Tumor Screening in Beckwith-Wiedemann Spectrum. J. Cancer Res. Clin. Oncol..

[41] Srinivasan A.S., Saade-Lemus S., Servaes S.E., Acord M.R., Reid J.R., Anupindi S.A., States L.J. (2019). Imaging Surveillance for Children with Predisposition to Renal Tumors. Pediatr. Radiol..

[42] Mussa A., Duffy K.A., Carli D., Ferrero G.B., Kalish J.M. (2019). Defining an Optimal Time Window to Screen for Hepatoblastoma in Children with Beckwith-Wiedemann Syndrome. Pediatr. Blood Cancer.

[43] Marshall G.M., Carter D.R., Cheung B.B., Liu T., Mateos M.K., Meyerowitz J.G., Weiss W.A. (2014). The Prenatal Origins of Cancer. Nat. Rev. Cancer.

[44] Knudson A.G. (1971). Mutation and Cancer: Statistical Study of Retinoblastoma. Proc. Natl. Acad. Sci. USA.

[45] Knudson A.G., Strong L.C. (1972). Mutation and Cancer: Neuroblastoma and Pheochromocytoma. Am. J. Hum. Genet..

[46] Knudson A.G., Strong L.C. (1972). Mutation and Cancer: A Model for Wilms’ Tumor of the Kidney. J. Natl. Cancer Inst..

[47] Hansford J.R., Das A., McGee R.B., Nakano Y., Brzezinski J., Scollon S.R., Rednam S.P., Schienda J., Michaeli O., Kim S.Y. (2024). Update on Cancer Predisposition Syndromes and Surveillance Guidelines for Childhood Brain Tumors. Clin. Cancer Res..

[48] Bakhuizen J.J., Hopman S.M.J., Bosscha M.I., Dommering C.J., van den Heuvel-Eibrink M.M., Hol J.A., Kester L.A., Koudijs M.J., Langenberg K.P.S., Loeffen J.L.C. (2023). Assessment of Cancer Predisposition Syndromes in a National Cohort of Children With a Neoplasm. JAMA Netw. Open.

[49] Kratz C.P., Jongmans M.C., Cavé H., Wimmer K., Behjati S., Guerrini-Rousseau L., Milde T., Pajtler K.W., Golmard L., Gauthier-Villars M. (2021). Predisposition to Cancer in Children and Adolescents. Lancet Child. Adolesc. Health.

[50] Fiala E.M., Jayakumaran G., Mauguen A., Kennedy J.A., Bouvier N., Kemel Y., Fleischut M.H., Maio A., Salo-Mullen E.E., Sheehan M. (2021). Prospective Pan-Cancer Germline Testing Using MSK-IMPACT Informs Clinical Translation in 751 Patients with Pediatric Solid Tumors. Nat. Cancer.

[51] Wilson C.L., Wang Z., Liu Q., Ehrhardt M.J., Mostafavi R., Easton J., Mulder H., Hedges D.J., Wang S., Rusch M. (2020). Estimated Number of Adult Survivors of Childhood Cancer in United States with Cancer-Predisposing Germline Variants. Pediatr. Blood Cancer.

[52] Simonin M., Vasseur L., Lengliné E., Lhermitte L., Cabannes-Hamy A., Balsat M., Schmidt A., Dourthe M.-E., Touzart A., Graux C. (2024). NGS-Based Stratification Refines the Risk Stratification in T-ALL and Identifies a Very-High-Risk Subgroup of Patients. Blood.

[53] Pulsipher M.A., Han X., Maude S.L., Laetsch T.W., Qayed M., Rives S., Boyer M.W., Hiramatsu H., Yanik G.A., Driscoll T. (2022). Next-Generation Sequencing of Minimal Residual Disease for Predicting Relapse after Tisagenlecleucel in Children and Young Adults with Acute Lymphoblastic Leukemia. Blood Cancer Discov..

[54] Wong M., Mayoh C., Lau L.M.S., Khuong-Quang D.-A., Pinese M., Kumar A., Barahona P., Wilkie E.E., Sullivan P., Bowen-James R. (2020). Whole Genome, Transcriptome and Methylome Profiling Enhances Actionable Target Discovery in High-Risk Pediatric Cancer. Nat. Med..

[55] Tsaousis G.N., Papadopoulou E., Apessos A., Agiannitopoulos K., Pepe G., Kampouri S., Diamantopoulos N., Floros T., Iosifidou R., Katopodi O. (2019). Analysis of Hereditary Cancer Syndromes by Using a Panel of Genes: Novel and Multiple Pathogenic Mutations. BMC Cancer.

[56] Helms L., Guimera A.E., Janeway K.A., Bailey K.M. (2023). Innovations in Cancer Treatment of Children. Pediatrics.

[57] Jahn A., Rump A., Widmann T.J., Heining C., Horak P., Hutter B., Paramasivam N., Uhrig S., Gieldon L., Drukewitz S. (2022). Comprehensive Cancer Predisposition Testing within the Prospective MASTER Trial Identifies Hereditary Cancer Patients and Supports Treatment Decisions for Rare Cancers. Ann. Oncol..

[58] Al-Sarhani H., Gottumukkala R.V., Grasparil A.D.S., Tung E.L., Gee M.S., Greer M.-L.C. (2022). Screening of Cancer Predisposition Syndromes. Pediatr. Radiol..

[59] Achatz M.I., Villani A., Bertuch A.A., Bougeard G., Chang V.Y., Doria A.S., Gallinger B., Godley L.A., Greer M.-L.C., Kamihara J. (2025). Update on Cancer Screening Recommendations for Individuals with Li-Fraumeni Syndrome. Clin. Cancer Res..

[60] Dhawan A., Baitamouni S., Liu D., Yehia L., Anthony K., McCarther A., Tischkowitz M., MacFarland S.P., Ngeow J., Hoogerbrugge N. (2025). Cancer and Overgrowth Manifestations of PTEN Hamartoma Tumor Syndrome: Management Recommendations from the International PHTS Consensus Guidelines Working Group. Clin. Cancer Res..

[61] Atluri H., Swaroop A., Godley L.A. (2025). Germline Predispositions to Myeloid Malignancies: Across the Lifespan. Semin. Hematol..

[62] Michaeli O., Kim S.Y., Mitchell S.G., Jongmans M.C.J., Wasserman J.D., Perrino M.R., Das A., MacFarland S.P., Scollon S.R., Greer M.-L.C. (2025). Update on Cancer Screening in Children with Syndromes of Bone Lesions, Hereditary Leiomyomatosis and Renal Cell Carcinoma Syndrome, and Other Rare Syndromes. Clin. Cancer Res..

[63] Nakano Y., Kuiper R.P., Nichols K.E., Porter C.C., Lesmana H., Meade J., Kratz C.P., Godley L.A., Maese L.D., Achatz M.I. (2024). Update on Recommendations for Cancer Screening and Surveillance in Children with Genomic Instability Disorders. Clin. Cancer Res..

[64] Maese L.D., Wlodarski M.W., Kim S.Y., Bertuch A.A., Bougeard G., Chang V.Y., Godley L.A., Khincha P.P., Kuiper R.P., Lesmana H. (2024). Update on Recommendations for Surveillance for Children with Predisposition to Hematopoietic Malignancy. Clin. Cancer Res..

[65] Vagher J., Mehrhoff C.J., Florou V., Maese L.D. (2024). Genetic Predisposition to Sarcoma: What Should Clinicians Know?. Curr. Treat. Options Oncol..

[66] Baruchel A., Bourquin J.-P., Crispino J., Cuartero S., Hasle H., Hitzler J., Klusmann J.-H., Izraeli S., Lane A.A., Malinge S. (2023). Down Syndrome and Leukemia: From Basic Mechanisms to Clinical Advances. Haematologica.

[67] Radtke H.B., Berger A., Skelton T., Goetsch Weisman A. (2023). Neurofibromatosis Type 1 (NF1): Addressing the Transition from Pediatric to Adult Care. Pediatr. Health Med. Ther..

[68] Rossini L., Durante C., Bresolin S., Opocher E., Marzollo A., Biffi A. (2022). Diagnostic Strategies and Algorithms for Investigating Cancer Predisposition Syndromes in Children Presenting with Malignancy. Cancers.

[69] Brioude F., Kalish J.M., Mussa A., Foster A.C., Bliek J., Ferrero G.B., Boonen S.E., Cole T., Baker R., Bertoletti M. (2018). Expert Consensus Document: Clinical and Molecular Diagnosis, Screening and Management of Beckwith-Wiedemann Syndrome: An International Consensus Statement. Nat. Rev. Endocrinol..

[70] Kamihara J., Bourdeaut F., Foulkes W.D., Molenaar J.J., Mossé Y.P., Nakagawara A., Parareda A., Scollon S.R., Schneider K.W., Skalet A.H. (2017). Retinoblastoma and Neuroblastoma Predisposition and Surveillance. Clin. Cancer Res..

[71] Achatz M.I., Porter C.C., Brugières L., Druker H., Frebourg T., Foulkes W.D., Kratz C.P., Kuiper R.P., Hansford J.R., Hernandez H.S. (2017). Cancer Screening Recommendations and Clinical Management of Inherited Gastrointestinal Cancer Syndromes in Childhood. Clin. Cancer Res..

[72] Ripperger T., Bielack S.S., Borkhardt A., Brecht I.B., Burkhardt B., Calaminus G., Debatin K.-M., Deubzer H., Dirksen U., Eckert C. (2017). Childhood Cancer Predisposition Syndromes-A Concise Review and Recommendations by the Cancer Predisposition Working Group of the Society for Pediatric Oncology and Hematology. Am. J. Med. Genet. A.

[73] Friedrich U.A., Bienias M., Zinke C., Prazenicova M., Lohse J., Jahn A., Menzel M., Langanke J., Walter C., Wagener R. (2023). A Clinical Screening Tool to Detect Genetic Cancer Predisposition in Pediatric Oncology Shows High Sensitivity but Can Miss a Substantial Percentage of Affected Children. Genet. Med..

[74] Samadder N.J., Riegert-Johnson D., Boardman L., Rhodes D., Wick M., Okuno S., Kunze K.L., Golafshar M., Uson P.L.S., Mountjoy L. (2021). Comparison of Universal Genetic Testing vs Guideline-Directed Targeted Testing for Patients With Hereditary Cancer Syndrome. JAMA Oncol..

[75] Wagener R., Taeubner J., Walter C., Yasin L., Alzoubi D., Bartenhagen C., Attarbaschi A., Classen C.-F., Kontny U., Hauer J. (2021). Comprehensive Germline-Genomic and Clinical Profiling in 160 Unselected Children and Adolescents with Cancer. Eur. J. Hum. Genet..

[76] Byrjalsen A., Hansen T.V.O., Stoltze U.K., Mehrjouy M.M., Barnkob N.M., Hjalgrim L.L., Mathiasen R., Lautrup C.K., Gregersen P.A., Hasle H. (2020). Nationwide Germline Whole Genome Sequencing of 198 Consecutive Pediatric Cancer Patients Reveals a High Incidence of Cancer Prone Syndromes. PLoS Genet..

[77] Goudie C., Cullinan N., Villani A., Mathews N., van Engelen K., Malkin D., Irwin M.S., Foulkes W.D. (2018). Retrospective Evaluation of a Decision-Support Algorithm (MIPOGG) for Genetic Referrals for Children with Neuroblastic Tumors. Pediatr. Blood Cancer.

[78] Goudie C., Coltin H., Witkowski L., Mourad S., Malkin D., Foulkes W.D. (2017). The McGill Interactive Pediatric OncoGenetic Guidelines: An Approach to Identifying Pediatric Oncology Patients Most Likely to Benefit from a Genetic Evaluation. Pediatr. Blood Cancer.

[79] Jongmans M.C.J., Loeffen J.L.C.M., Waanders E., Hoogerbrugge P.M., Ligtenberg M.J.L., Kuiper R.P., Hoogerbrugge N. (2016). Recognition of Genetic Predisposition in Pediatric Cancer Patients: An Easy-to-Use Selection Tool. Eur. J. Med. Genet..

