# Supplementary material for: Epidemiology of Childhood Cancer and Cancer Predisposition Syndromes (CPSs): A 20-Year Single-Center Cohort from the Greater Poland Region
Source: Children (Basel). 2026 Jun 3;13(6):778. doi: 10.3390/children13060778 (PMC13297731; doi:10.3390/children13060778)
Supplement: Supplementary file 1 [file children-13-00778-s001.zip › Supplementary Table S1.pdf]

Supplementary Table S1. The list of all cancer predisposition syndromes (CPSs) and related clinical features observed among the group of 193 CPS-positive patients. Highlighted CPSs (*) were confirmed with any kind of genetic testing in patients from the exact CPS group. In case of dysmorphic features and isolated hemihypertrophy (IHH), the patient was recognized as CPS-positive by one's clinical symptoms only. All children diagnosed with Beckwith-Widemann Syndrome (BWS) and neurofibromatosis type 1 (NF1) presented typical clinical features for each syndrome, but not every one of them was confirmed in genetic testing.

Cancer predisposition syndrome (CPS)	Genetic and/or clinical findings
Beckwith-Widemann Syndrome (BWS)	11p15 alterations and/or hemihypertrophy, macrosomia, macroglossia, umbilical hernia
BRCA gene mutation*	BRCA1 gene mutation
Constitutional Mismatch Repair Deficiency Syndrome (CMMRD)*	Patient 1: co-occurrence of MSH2 and MSH6 gene mutations, Patient 2: co-occurrence of MSH2 and PMS2 gene mutations, and IgA deficiency, café-au-lait spots, FASI changes in the central nervous system MRI
DICER1 gene mutation*	DICER1 gene mutations and thyroid multinodular goiter in the female family line
Down Syndrome (DS)*	Trisomy 21 and its clinical features
Dysmorphic features	Symptoms combined of: developmental delay, short stature, joint hypermobility, decreased muscle tone, hemihypertrophy, macrocephaly, microcephaly, low-set ears, tall forehead, hypertelorism, epicanthal folds, macroglossia, dental anomalies, short nose, small chin, short neck, café-au-lait spots, skin hypopigmentation, vascular changes, photosensitivity
Edwards' Syndrome (ES)*	Trisomy 18 (mosaic) and its clinical features
Fanconi Anemia (FA)*	BRCA2 (FANCD1) gene mutation and short stature, café-au-lait spots, horseshoe kidney
Hemophagocytic lymphohistiocytosis (HLH)*	UNC13D or STX11 gene mutations and laboratory features
Hirschsprung disease (HSCR)*	Both HSCR and PHOX2B gene mutations
Isolated hemihypertrophy (IHH)	Hemihypertrophy
IMAGE Syndrome*	CDKN1C gene mutation and its clinical features
Li-Fraumeni Syndrome (Li-FS)*	TP53 gene mutations
Multiple Endocrine Neoplasia Syndrome (MEN)*	RET gene mutations
Neurofibromatosis type 1 (NF1)	NF1 gene mutations and/or 5 or more café-au-lait spots in the patient, 5 or more café-au-lait spots in the patient's close family members (one of the parents, siblings), 2 or more neurofibromas of any type, optic pathway glioma
Nijmegen Breakage Syndrome (NBS)*	NBS1 gene mutation and its clinical features
Noonan Syndrome*	Co-occurrence of PTPN11, KRAS, and MEK1 gene mutations, and its clinical features
Rhabdoid Tumor Predisposition Syndrome (RTPS)*	SMARCB1 gene mutations
Von Hippel-Lindau Syndrome (VHL)*	VHL gene mutation
WAGR Syndrome*	11p deletion and its clinical features
