# Supplementary material for: Epidemiology of Childhood Cancer and Cancer Predisposition Syndromes (CPSs): A 20-Year Single-Center Cohort from the Greater Poland Region
Source: Children (Basel). 2026 Jun 3;13(6):778. doi: 10.3390/children13060778 (PMC13297731; doi:10.3390/children13060778)
Supplement: Supplementary file 1 [file children-13-00778-s001.zip › Supplementary Table S2.pdf]

*Supplementary Table S2. Chi-square goodness-of-fit test results comparing observed and expected age distributions across ICD-10 categories. Raw and Benjamini-Hochberg FDR-adjusted p-values are shown.*

| ICD-10 code | $p$ | $P_{FDR}$ |
|---|---|---|
| C22 | <0.001 | <0.001 |
| C38 | <0.001 | <0.001 |
| C39 | <0.001 | <0.001 |
| C41 | <0.001 | <0.001 |
| C47 | <0.001 | <0.001 |
| C48 | <0.001 | <0.001 |
| C49 | <0.001 | <0.001 |
| C62 | <0.001 | <0.001 |
| C64 | <0.001 | <0.001 |
| C67 | <0.001 | <0.001 |
| C68 | <0.001 | <0.001 |
| C69 | <0.001 | <0.001 |
| C70 | <0.001 | <0.001 |
| C71 | <0.001 | <0.001 |
| C73 | <0.001 | <0.001 |
| C81 | <0.001 | <0.001 |
| C91 | <0.001 | <0.001 |
| C92 | <0.001 | <0.001 |
| C96 | <0.001 | <0.001 |
| C76 | 0.012 | 0.023 |
| C30 | 0.033 | 0.059 |
| C40 | 0.193 | 0.324 |
| C83 | 0.332 | 0.496 |
| C37 | 0.335 | 0.496 |
| C84 | 0.335 | 0.496 |
| C56 | 0.354 | 0.504 |
| C85 | 0.607 | 0.832 |
| C80 | 0.706 | 0.933 |
| C79 | 0.820 | 0.991 |
| C74 | 0.837 | 0.991 |
| C94 | 0.865 | 0.991 |
| C11 | 0.887 | 0.991 |
| C75 | 0.894 | 0.991 |
| C95 | 0.925 | 0.991 |
| C88 | 0.976 | 0.991 |
| C43 | 0.987 | 0.991 |
| C72 | 0.991 | 0.991 |
