# Supplementary material for: Epidemiology of Childhood Cancer and Cancer Predisposition Syndromes (CPSs): A 20-Year Single-Center Cohort from the Greater Poland Region
Source: Children (Basel). 2026 Jun 3;13(6):778. doi: 10.3390/children13060778 (PMC13297731; doi:10.3390/children13060778)
Supplement: Supplementary file 1 [file children-13-00778-s001.zip › Supplementary Table S3.pdf]

Supplementary Table S3. Age-specific RR estimates with 95% confidence intervals for all ICD-10 categories. The reference group was defined as the youngest age group with at least five observed cases; when no such group was available, the age group with the highest number of cases was used as the reference. Estimates based on fewer than five cases are considered unstable. Abbreviations: d.o.: days old, m.o.: months old, y.o.: years old.

ICD-10 code	Age group	Cases	RR (95% CI)	Stability
C11	0-28 d.o.	0	–	unstable
	1-2 m.o.	0	–	unstable
	3-11 m.o.	0	–	unstable
	1-2 y.o.	1	2.436 (0.221-26.868)	unstable
	3-6 y.o.	0	–	unstable
	**7-15 y.o.**	**2**	**1.000 (1.000-1.000)**	**reference**
	16-17 y.o.	1	1.991 (0.181-21.961)	unstable
C22	0-28 d.o.	0	–	unstable
	1-2 m.o.	4	2.571 (0.753-8.784)	unstable
	**3-11 m.o.**	**7**	**1.000 (1.000-1.000)**	**reference**
	1-2 y.o.	14	0.744 (0.300-1.844)	stable
	3-6 y.o.	7	0.185 (0.065-0.528)	stable
	7-15 y.o.	9	0.098 (0.037-0.264)	stable
	16-17 y.o.	1	0.043 (0.005-0.353)	unstable
C38	0-28 d.o.	2	12.094 (2.215-66.033)	unstable
	1-2 m.o.	0	–	unstable
	3-11 m.o.	0	–	unstable
	**1-2 y.o.**	**4**	**1.000 (1.000-1.000)**	**reference**
	3-6 y.o.	0	–	unstable
	7-15 y.o.	1	0.051 (0.006-0.459)	unstable
	16-17 y.o.	0	–	unstable
C40	0-28 d.o.	0	–	unstable
	1-2 m.o.	0	–	unstable
	3-11 m.o.	0	–	unstable
	1-2 y.o.	0	–	unstable
	**3-6 y.o.**	**7**	**1.000 (1.000-1.000)**	**reference**
	7-15 y.o.	22	1.295 (0.553-3.032)	stable
	16-17 y.o.	8	1.876 (0.680-5.173)	stable
C41	0-28 d.o.	2	19.643 (4.396-87.768)	unstable
	1-2 m.o.	0	–	unstable
	3-11 m.o.	3	3.274 (0.924-11.601)	unstable
	1-2 y.o.	3	1.218 (0.344-4.317)	unstable
	3-6 y.o.	3	0.607 (0.171-2.150)	unstable
	**7-15 y.o.**	**12**	**1.000 (1.000-1.000)**	**reference**
	16-17 y.o.	4	1.328 (0.428-4.116)	unstable
C47	**0-28 d.o.**	**18**	**1.000 (1.000-1.000)**	**reference**
	1-2 m.o.	14	0.389 (0.193-0.782)	stable
	3-11 m.o.	32	0.198 (0.111-0.352)	stable
	1-2 y.o.	52	0.119 (0.070-0.204)	stable
	3-6 y.o.	38	0.043 (0.025-0.076)	stable
	7-15 y.o.	19	0.009 (0.005-0.017)	stable
	16-17 y.o.	4	0.008 (0.003-0.022)	unstable
C48	**0-28 d.o.**	**1**	**1.000 (1.000-1.000)**	**reference**
	1-2 m.o.	0	–	unstable
	3-11 m.o.	0	–	unstable
	1-2 y.o.	0	–	unstable
	3-6 y.o.	1	0.021 (0.001-0.329)	unstable
	7-15 y.o.	1	0.008 (0.001-0.136)	unstable

	16-17 y.o.	1	0.034 (0.002-0.540)	unstable
C49	**0-28 d.o.**	**5**	**1.000 (1.000-1.000)**	**reference**
	1-2 m.o.	10	1.000 (0.342-2.926)	stable
	3-11 m.o.	16	0.356 (0.130-0.971)	stable
	1-2 y.o.	33	0.273 (0.107-0.699)	stable
	3-6 y.o.	35	0.144 (0.056-0.368)	stable
	7-15 y.o.	45	0.076 (0.030-0.192)	stable
	16-17 y.o.	17	0.115 (0.042-0.311)	stable
C56	0-28 d.o.	0	–	unstable
	1-2 m.o.	0	–	unstable
	3-11 m.o.	1	0.818 (0.109-6.172)	unstable
	1-2 y.o.	0	–	unstable
	3-6 y.o.	2	0.303 (0.070-1.319)	unstable
	**7-15 y.o.**	**16**	**1.000 (1.000-1.000)**	**reference**
	16-17 y.o.	2	0.498 (0.114-2.165)	unstable
C62	0-28 d.o.	0	–	unstable
	1-2 m.o.	0	–	unstable
	3-11 m.o.	2	0.896 (0.181-4.439)	unstable
	**1-2 y.o.**	**6**	**1.000 (1.000-1.000)**	**reference**
	3-6 y.o.	1	0.083 (0.010-0.689)	unstable
	7-15 y.o.	5	0.171 (0.052-0.560)	stable
	16-17 y.o.	9	1.226 (0.436-3.445)	stable
C64	0-28 d.o.	3	1.929 (0.554-6.711)	unstable
	1-2 m.o.	0	–	unstable
	**3-11 m.o.**	**14**	**1.000 (1.000-1.000)**	**reference**
	1-2 y.o.	29	0.771 (0.407-1.459)	stable
	3-6 y.o.	61	0.807 (0.452-1.443)	stable
	7-15 y.o.	16	0.087 (0.043-0.179)	stable
	16-17 y.o.	7	0.152 (0.061-0.377)	stable
C69	0-28 d.o.	0	–	unstable
	1-2 m.o.	0	–	unstable
	3-11 m.o.	4	4.317 (1.159-16.078)	unstable
	1-2 y.o.	3	1.205 (0.288-5.041)	unstable
	**3-6 y.o.**	**5**	**1.000 (1.000-1.000)**	**reference**
	7-15 y.o.	0	–	unstable
	16-17 y.o.	0	–	unstable
C70	0-28 d.o.	0	–	unstable
	1-2 m.o.	1	6.047 (0.548-66.693)	unstable
	3-11 m.o.	0	–	unstable
	**1-2 y.o.**	**2**	**1.000 (1.000-1.000)**	**reference**
	3-6 y.o.	0	–	unstable
	7-15 y.o.	2	0.205 (0.029-1.457)	unstable
	16-17 y.o.	0	–	unstable
C71	**0-28 d.o.**	**7**	**1.000 (1.000-1.000)**	**reference**
	1-2 m.o.	4	0.286 (0.084-0.976)	unstable
	3-11 m.o.	21	0.333 (0.142-0.784)	stable
	1-2 y.o.	58	0.343 (0.156-0.750)	stable
	3-6 y.o.	96	0.282 (0.131-0.608)	stable
	7-15 y.o.	134	0.162 (0.076-0.347)	stable
	16-17 y.o.	37	0.179 (0.080-0.401)	stable
C72	0-28 d.o.	0	–	unstable
	1-2 m.o.	0	–	unstable
	3-11 m.o.	1	1.310 (0.168-10.230)	unstable

	1-2 y.o.	3	1.462 (0.402-5.311)	unstable
	3-6 y.o.	4	0.971 (0.304-3.095)	unstable
	**7-15 y.o.**	**10**	**1.000 (1.000-1.000)**	**reference**
	16-17 y.o.	2	0.797 (0.175-3.635)	unstable
C73	0-28 d.o.	0	–	unstable
	1-2 m.o.	0	–	unstable
	3-11 m.o.	0	–	unstable
	1-2 y.o.	0	–	unstable
	3-6 y.o.	2	0.124 (0.030-0.515)	unstable
	**7-15 y.o.**	**39**	**1.000 (1.000-1.000)**	**reference**
	16-17 y.o.	32	3.268 (2.047-5.215)	stable
C74	0-28 d.o.	0	–	unstable
	1-2 m.o.	0	–	unstable
	3-11 m.o.	1	1.079 (0.126-9.239)	unstable
	1-2 y.o.	3	1.205 (0.288-5.041)	unstable
	**3-6 y.o.**	**5**	**1.000 (1.000-1.000)**	**reference**
	7-15 y.o.	7	0.577 (0.183-1.818)	stable
	16-17 y.o.	1	0.328 (0.038-2.810)	unstable
C75	0-28 d.o.	0	–	unstable
	1-2 m.o.	0	–	unstable
	3-11 m.o.	0	–	unstable
	1-2 y.o.	0	–	unstable
	3-6 y.o.	1	0.607 (0.068-5.428)	unstable
	**7-15 y.o.**	**4**	**1.000 (1.000-1.000)**	**reference**
	16-17 y.o.	0	–	unstable
C76	0-28 d.o.	0	–	unstable
	1-2 m.o.	0	–	unstable
	3-11 m.o.	3	7.857 (1.878-32.878)	unstable
	1-2 y.o.	3	2.923 (0.699-12.233)	unstable
	3-6 y.o.	1	0.485 (0.057-4.154)	unstable
	**7-15 y.o.**	**5**	**1.000 (1.000-1.000)**	**reference**
	16-17 y.o.	1	0.797 (0.093-6.818)	unstable
C81	0-28 d.o.	0	–	unstable
	1-2 m.o.	0	–	unstable
	3-11 m.o.	1	0.540 (0.069-4.216)	unstable
	1-2 y.o.	0	–	unstable
	**3-6 y.o.**	**10**	**1.000 (1.000-1.000)**	**reference**
	7-15 y.o.	88	3.627 (1.886-6.975)	stable
	16-17 y.o.	45	7.386 (3.722-14.655)	stable
C83	0-28 d.o.	1	2.419 (0.310-18.896)	unstable
	1-2 m.o.	0	–	unstable
	3-11 m.o.	2	0.538 (0.118-2.453)	unstable
	**1-2 y.o.**	**10**	**1.000 (1.000-1.000)**	**reference**
	3-6 y.o.	27	1.345 (0.651-2.778)	stable
	7-15 y.o.	73	1.498 (0.774-2.901)	stable
	16-17 y.o.	11	0.899 (0.382-2.117)	stable
C85	0-28 d.o.	0	–	unstable
	1-2 m.o.	0	–	unstable
	3-11 m.o.	0	–	unstable
	1-2 y.o.	0	–	unstable
	3-6 y.o.	3	1.820 (0.407-8.132)	unstable
	**7-15 y.o.**	**4**	**1.000 (1.000-1.000)**	**reference**
	16-17 y.o.	3	2.987 (0.668-13.346)	unstable

C88	0-28 d.o.	0	–	unstable
	1-2 m.o.	0	–	unstable
	3-11 m.o.	0	–	unstable
	1-2 y.o.	1	1.218 (0.136-10.899)	unstable
	3-6 y.o.	3	1.820 (0.407-8.132)	unstable
	**7-15 y.o.**	**4**	**1.000 (1.000-1.000)**	**reference**
	16-17 y.o.	1	0.996 (0.111-8.908)	unstable
C91	0-28 d.o.	0	–	unstable
	1-2 m.o.	4	2.000 (0.616-6.495)	unstable
	**3-11 m.o.**	**9**	**1.000 (1.000-1.000)**	**reference**
	1-2 y.o.	90	3.721 (1.875-7.383)	stable
	3-6 y.o.	187	3.850 (1.972-7.515)	stable
	7-15 y.o.	188	1.595 (0.817-3.114)	stable
	16-17 y.o.	27	0.912 (0.429-1.940)	stable
C92	**0-28 d.o.**	**5**	**1.000 (1.000-1.000)**	**reference**
	1-2 m.o.	0	–	unstable
	3-11 m.o.	4	0.089 (0.024-0.331)	unstable
	1-2 y.o.	30	0.248 (0.096-0.639)	stable
	3-6 y.o.	30	0.124 (0.048-0.318)	stable
	7-15 y.o.	94	0.160 (0.065-0.392)	stable
	16-17 y.o.	24	0.162 (0.062-0.425)	stable
C94	0-28 d.o.	0	–	unstable
	1-2 m.o.	0	–	unstable
	3-11 m.o.	0	–	unstable
	1-2 y.o.	1	0.696 (0.086-5.658)	unstable
	3-6 y.o.	2	0.693 (0.144-3.337)	unstable
	**7-15 y.o.**	**7**	**1.000 (1.000-1.000)**	**reference**
	16-17 y.o.	0	–	unstable
C96	0-28 d.o.	0	–	unstable
	1-2 m.o.	0	–	unstable
	**3-11 m.o.**	**10**	**1.000 (1.000-1.000)**	**reference**
	1-2 y.o.	19	0.707 (0.329-1.520)	stable
	3-6 y.o.	6	0.111 (0.040-0.306)	stable
	7-15 y.o.	13	0.099 (0.044-0.226)	stable
	16-17 y.o.	1	0.030 (0.004-0.238)	unstable
